# DONSON-Associated MISSLA with Co-Occurring p.Arg442Ter and p.Lys489Thr Variants: Longitudinal Clinical and Genomic Characterization

**DOI:** 10.3390/genes17101211

**Published:** 2026-09-29

**Authors:** Fabricio González-Andrade

**Affiliations:** Dean’s Office of Graduate Studies in Health Sciences, Faculty of Health and Welfare Sciences, Indoamerica Technological University, Machala and Sabanilla Street, Quito 170301, Ecuador; fabriciogonzaleza@gmail.com or fabriciogonzalez@uti.edu.ec

**Keywords:** DONSON, MISSLA, microcephaly, primordial dwarfism, p.Arg442Ter, p.Lys489Thr, nonsense-mediated mRNA decay, NMDetective-B, growth restriction, longitudinal phenotype

## Abstract

**Background**: Microcephaly, short stature, and limb abnormalities syndrome (MISSLA) is an ultra-rare developmental disorder associated with biallelic DONSON variation and characterized by prenatal-onset growth restriction, congenital microcephaly, variable skeletal abnormalities, and neurodevelopmental impairment. We describe the longitudinal clinical and genomic features of an Ecuadorian child with a phenotype highly compatible with DONSON-associated MISSLA. **Methods**: This retrospective longitudinal case report included clinical information from the prenatal period through February 2026. Serial anthropometric, neurodevelopmental, nutritional, skeletal, and neuroimaging findings were reviewed. Clinical whole-exome sequencing with mitochondrial DNA analysis was performed during infancy. DONSON variants were evaluated using current transcript nomenclature, population and clinical databases, and published variant-specific evidence. Because patient-derived RNA was unavailable, the predicted susceptibility of NM_017613.4:c.1324C>T (p.Arg442Ter) to nonsense-mediated mRNA decay (NMD) was assessed using NMDetective-B. **Results**: The proband had severe prenatal growth restriction and congenital microcephaly, followed by persistent profound short stature, marked postnatal weight impairment, feeding difficulties, developmental delay with continued acquisition of motor and language abilities, and relatively mild skeletal involvement. At 7 years and 4 months, weight was 10.3 kg (Z = −8.02) and height was 91.5 cm (Z = −5.00). Whole-exome sequencing identified heterozygous DONSON c.1324C>T (p.Arg442Ter) and c.1466A>C (p.Lys489Thr), together with c.82A>C (p.Ser28Arg). The recurrent intronic c.786-33A>G variant was not detected, although limited coverage precluded definitive exclusion. Parental segregation data were unavailable, and compound heterozygosity was therefore not established. NMDetective-B yielded a score of 0.65, predicting that p.Arg442Ter is susceptible to NMD. **Conclusions**: This case extends the longitudinal phenotypic characterization of DONSON-associated MISSLA and documents a distinct co-occurring variant configuration. The NMD result provides a testable transcript-level hypothesis for p.Arg442Ter, but the absence of parental phasing and patient-derived RNA or protein studies precludes definitive assignment of a patient-specific molecular mechanism.

## 1. Introduction

Microcephaly, short stature, and limb abnormalities syndrome (MISSLA; OMIM #617604) is an ultra-rare developmental disorder within the microcephalic primordial dwarfism spectrum associated with pathogenic variation in DONSON. The official gene name is DNA replication fork stabilization factor DONSON, and the gene is located on chromosome 21q22.11 [1].

The association between DONSON and human developmental disease was established by Reynolds et al., who identified recessive DONSON variants in individuals with severe prenatal and postnatal growth restriction, microcephaly, and variable skeletal abnormalities [2]. In the same study, patient-derived cellular models demonstrated increased spontaneous replication-fork stalling and impaired genome maintenance, providing the first direct functional evidence linking DONSON deficiency to human microcephalic dwarfism [2].

The clinical spectrum was subsequently refined by the description of biallelic DONSON variants in individuals with microcephaly, severe short stature, and limb abnormalities, establishing the phenotype now recognized as MISSLA [3]. A homozygous deep-intronic DONSON variant causing abnormal RNA splicing was independently identified in microcephaly–micromelia syndrome, demonstrating that severe developmental disease can also result from disruption of DONSON expression by a noncoding variant [4]. Broader clinical studies subsequently demonstrated substantial allelic and phenotypic heterogeneity, including microcephalic dwarfism, Seckel-like manifestations, skeletal abnormalities, and Meier–Gorlin-like presentations [5].

More recent experimental work has clarified the role of DONSON in DNA-replication initiation. DONSON participates directly in assembly of the CDC45–MCM2-7–GINS (CMG) replicative helicase, establishing a mechanistic connection between DONSON deficiency and impaired origin activation [6]. These experimental observations provide a biological framework for understanding severe developmental growth restriction in DONSON-associated disorders, although they should not be interpreted as patient-specific functional findings in the absence of corresponding cellular studies.

Of particular relevance to MISSLA is DONSON NM_017613.4:c.1466A>C (p.Lys489Thr). Reynolds et al. identified p.Lys489Thr recurrently within an allele containing c.82A>C (p.Ser28Arg), c.786-33A>G, and c.1466A>C (p.Lys489Thr) [2]. Functional experiments showed that p.Lys489Thr was associated with reduced DONSON protein abundance and incomplete correction of spontaneous replication-fork stalling [2]. In contrast, p.Ser28Arg did not reproduce the same reduction in protein abundance, and the evaluated c.786-33A>G substitution did not demonstrate the initially proposed splice-disrupting effect [2]. These observations emphasize that the biological properties attributed to a multi-variant DONSON allele should not automaticallssy be assigned to each component sequence change individually.

The second principal variant identified in the present case, DONSON NM_017613.4:c.1324C>T (p.Arg442Ter), is a nonsense substitution that introduces a premature termination codon at amino acid position 442. The original clinical diagnostic laboratory classified this variant as pathogenic. Reynolds et al. previously reported other truncating variants in the same general region of DONSON, including p.Lys419Ter and p.Gln428Ter, establishing disease-associated precedent for premature termination variants affecting this portion of the protein [2]. However, p.Arg442Ter itself was not reported among the clinically characterized individuals in that foundational cohort [2].

The present study concerns an Ecuadorian male child with severe prenatal-onset growth restriction, congenital microcephaly, persistent profound short stature, developmental impairment with progressive acquisition of functional abilities, feeding and nutritional difficulties, and relatively mild limb abnormalities. Whole-exome sequencing identified heterozygous DONSON c.1324C>T (p.Arg442Ter) and c.1466A>C (p.Lys489Thr), together with c.82A>C (p.Ser28Arg). The recurrent intronic c.786-33A>G variant was not detected by the clinical assay; however, limited sequencing coverage of this region prevents its definitive exclusion. Parental segregation testing was recommended in the original molecular report, but no formal result demonstrating that the principal variants are in trans was identified in the available records. The clinical documentation therefore supports a clinical molecular diagnosis highly compatible with DONSON-associated MISSLA, while confirmation of the biallelic configuration remains pending.

The availability of longitudinal clinical records from fetal life through school age permits analysis of the natural history of the phenotype rather than characterization at a single time point. Severe linear growth restriction was already established at birth, with length approximately −4.92 SD, and remained of similar magnitude at 7 years and 4 months, when height was approximately −5.00 SD. In contrast, weight changed from approximately −3.52 SD at birth to −8.02 SD at 7 years and 4 months, indicating disproportionate postnatal deterioration of body weight in the context of documented feeding and nutritional difficulties. This distinction permits the persistent primordial growth phenotype to be considered separately from a superimposed nutritional component, an interpretation also supported by the longitudinal clinical assessment.

Accordingly, the objective of the present study was to perform an integrated longitudinal clinical and variant-level genomic characterization of this patient. We specifically aimed to characterize the evolution of growth, neurodevelopment, nutritional manifestations, and skeletal findings from fetal life through school age; define the observed DONSON sequence configuration without assuming unconfirmed phase; evaluate variant-specific evidence relevant to p.Lys489Thr; and assess the predicted susceptibility of p.Arg442Ter to nonsense-mediated mRNA decay using the transcript-specific NMDetective-B framework. By separating original patient-derived and computational findings from literature-based mechanistic context, the study was designed to provide a more reproducible and appropriately bounded interpretation of this ultra-rare DONSON-associated phenotype.

## 2. Case Presentation and Methods

### 2.1. Clinical Ascertainment and Longitudinal Phenotyping

This retrospective longitudinal case report describes a male child born in Ecuador and followed from the prenatal period through February 2026. Clinical information was obtained from the longitudinal medical record of the Carlos Andrade Marín Specialty Hospital (HECAM), Ecuadorian Social Security Institute (IESS), Quito, Ecuador, covering the period from 2018 to 2026. The reviewed documentation included prenatal and neonatal records, pediatric and medical genetics evaluations, neurologic and neurodevelopmental assessments, endocrinology and nutrition consultations, rehabilitation and ophthalmology evaluations, conventional cytogenetic testing, laboratory investigations, fetal and postnatal neuroimaging reports, and the original clinical whole-exome sequencing report. Only findings explicitly documented in the available medical records were included.

Clinical information was reviewed chronologically to characterize the evolution of prenatal growth, birth anthropometry, postnatal growth, neurodevelopment, neurologic manifestations, skeletal findings, feeding and nutrition, endocrine findings, and neuroimaging. Findings reported in isolated assessments without confirmation elsewhere in the longitudinal record were not considered established manifestations.

Serial weight, length or height, head circumference, and body mass index measurements were extracted from the clinical record. When Z scores had been calculated contemporaneously by the treating clinical services, these values were retained rather than retrospectively replaced. Measurements from birth through 5 years of age were evaluated using the World Health Organization Child Growth Standards [7], whereas measurements after 5 years were interpreted using the WHO 2007 Growth Reference for school-aged children and adolescents [8]. Weight and linear-growth trajectories were evaluated separately to distinguish persistent prenatal-onset growth restriction from disproportionate postnatal weight impairment associated with feeding and nutritional morbidity. Developmental progression was reconstructed from pediatric, neurologic, rehabilitation, neuropsychological, and educational assessments, with particular attention to gross-motor development, language, adaptive function, and school performance.

### 2.2. Genetic Testing and Variant Interpretation

Clinical whole-exome sequencing (WES) with mitochondrial DNA analysis was performed during infancy using a dried-blood-spot sample. The diagnostic report was issued in 2019. According to the original laboratory documentation, the assay comprised an approximately 60 Mb exome analysis covering approximately 20,000 genes together with mitochondrial DNA assessment. DNA underwent extraction and quality assessment, library preparation, and paired-end sequencing using an Illumina-based workflow. The targeted exonic content comprised approximately 48 Mb, and sequence reads were aligned to the GRCh37/hg19 human reference assembly. Mitochondrial sequences were assessed using off-target reads generated during exome sequencing. The reported mean on-target bait coverage was 133.43×, with approximately 99.04% of targeted bases covered at ≥10× and 97.18% at ≥20×. The laboratory indicated that large deletions or duplications, repeat expansions, and variants in inadequately covered or technically difficult regions could be incompletely detected.

The original diagnostic report identified heterozygous DONSON c.1324C>T (p.Arg442Ter), classified as pathogenic, and c.1466A>C (p.Lys489Thr), classified as likely pathogenic. Heterozygous c.82A>C (p.Ser28Arg) was also documented. The recurrent intronic c.786-33A>G sequence change was reported as not detected; however, because coverage of this region was limited, non-detection was not considered definitive exclusion.

The original report used transcript NM_017613.3. For the present analysis, variant nomenclature was harmonized to the current MANE Select transcript NM_017613.4 (ENST00000303071.10) [9]. The nonsense variant originally reported as p.Arg442* is expressed throughout the manuscript as p.Arg442Ter, an equivalent HGVS stop-codon designation. Variant positions were evaluated relative to the 566-amino-acid DONSON protein.

Because DONSON-associated MISSLA is predominantly associated with biallelic variation, available records were specifically reviewed for evidence of parental segregation. Parental testing had been recommended by the diagnostic laboratory, but no formal molecular result demonstrating that p.Arg442Ter and p.Lys489Thr occur on opposite alleles was identified. The variants were therefore treated as co-occurring heterozygous variants, and compound heterozygosity or a trans configuration was not assumed.

Contemporary variant information was reviewed separately from the original 2019 laboratory classification. ClinVar was examined for variant-specific clinical interpretations and disease associations [10], and the Genome Aggregation Database (gnomAD), version 4.1, was queried for population allele frequencies [11]. Particular attention was given to p.Lys489Thr because it is present in population datasets and has previously been reported as part of a recurrent DONSON allele containing p.Ser28Arg, c.786-33A>G, and p.Lys489Thr. Published functional observations were attributed only to the specific variants or experimental configurations in which they had been demonstrated and were not treated as patient-specific experimental findings.

A targeted PubMed review was performed through August 2026 to identify molecularly characterized DONSON-associated disorders and reports relevant to p.Arg442Ter, p.Lys489Thr, p.Ser28Arg, and c.786-33A>G. The review was used to determine whether the observed variants or related sequence configurations had previously been described in affected individuals and to identify relevant experimentally demonstrated variant-specific effects. It was intended for contextual interpretation and was not conducted as a systematic review or meta-analysis.

### 2.3. In Silico Nonsense-Mediated mRNA Decay Analysis and Protein Context

Because patient-derived RNA was unavailable, the susceptibility of DONSON NM_017613.4:c.1324C>T (p.Arg442Ter) to nonsense-mediated mRNA decay (NMD) was assessed in silico using the published NMDetective-B framework [12]. The analysis used the MANE Select transcript NM_017613.4 (ENST00000303071.10). The premature termination codon was evaluated according to the four transcript-position determinants incorporated in the NMDetective-B decision model: localization within the last exon, localization within the terminal 50 nucleotides of the penultimate exon, proximity to the translation-initiation codon defined as fewer than 150 coding nucleotides, and localization within a coding exon exceeding 407 nucleotides [12]. Transcript structure, coding-exon position, and coding distance from the translation-initiation site were evaluated before assignment of the corresponding terminal branch of the decision model.

The NMDetective-B output was interpreted solely as a computational prediction of NMD susceptibility. It was not considered evidence of experimentally demonstrated degradation of the mutant transcript, altered allelic expression, reduced patient RNA abundance, or reduced DONSON protein expression.

Protein-level positional context was assessed using the reviewed human DONSON entry in UniProtKB (Q9NYP3) [13]. Because curated annotation does not define a well-established discrete functional subdomain specifically encompassing residues 442 and 489, variant interpretation was based primarily on their positions within the 566-amino-acid protein and on previously reported disease-associated variants rather than on speculative domain assignments.

### 2.4. Ethics and Consent

The clinical case and associated analysis were approved by the UTI Institutional Committee for Academic Review (CIRA) under approval No. UTI-DPS-CIRA-42-2026, dated 1 June 2026. The study was conducted in accordance with applicable institutional ethical requirements and the principles of the Declaration of Helsinki. Clinical and genomic information was restricted to data required for scientific characterization of the case, and direct personal identifiers were excluded from the manuscript. Medical records remained confidential and were reviewed only for the approved clinical and research purposes. Consent for publication was obtained in accordance with the institutional approval process.

### 2.5. Declaration on the Use of Generative Artificial Intelligence

Generative AI tools, including ChatGPT 5 (OpenAI), were used to assist with language editing, manuscript restructuring, and refinement of graphical elements. All clinical data, genomic findings, numerical results, references, and scientific interpretations were independently verified by the author, who takes full responsibility for the final content.

## 3. Results/Case Findings

**Figure 1:**
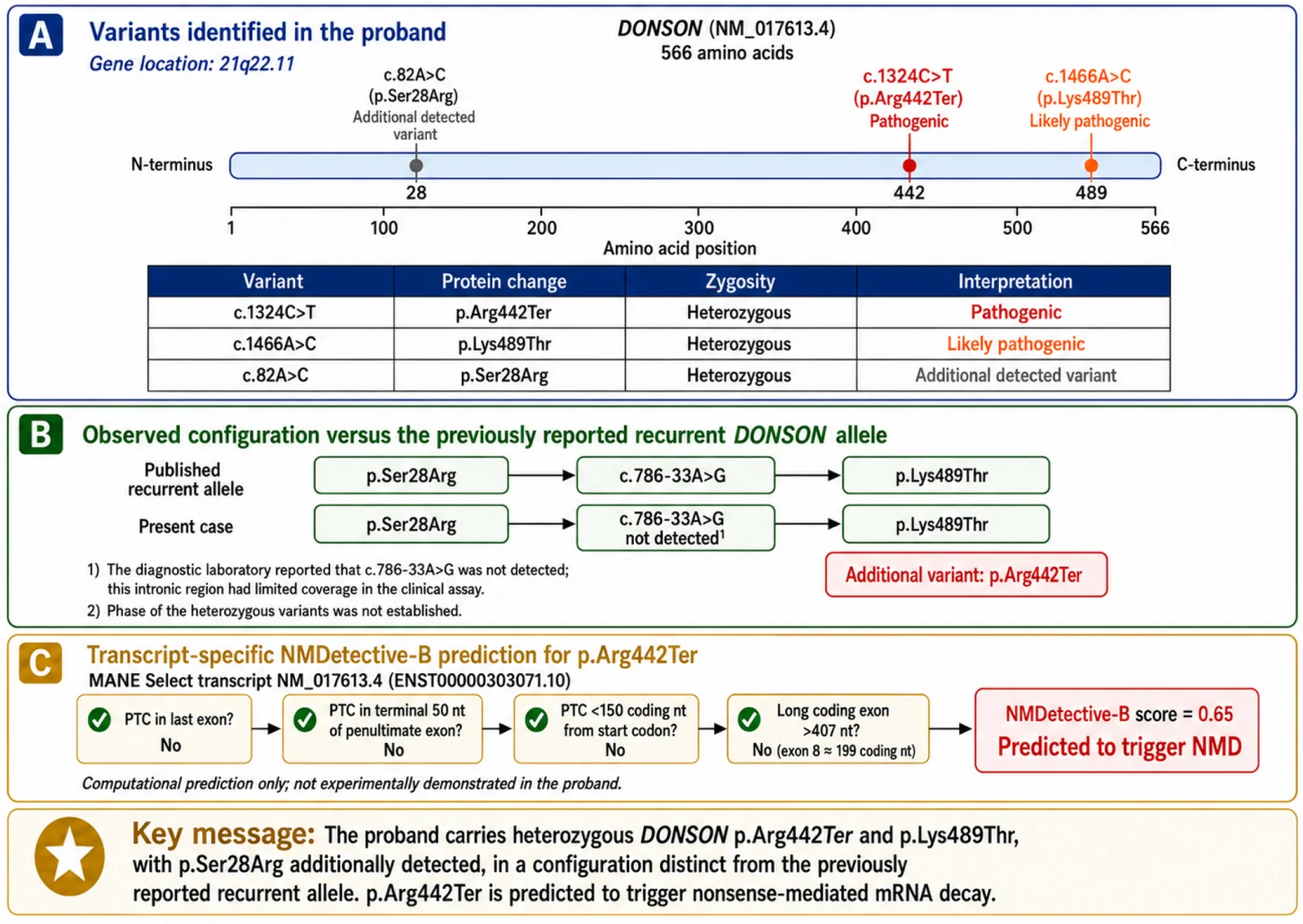
Integrated genomic findings, allelic context, and predicted nonsense-mediated mRNA decay susceptibility of DONSON p.Arg442Ter. (**A**) Schematic representation of the DONSON protein encoded by transcript NM_017613.4 (566 amino acids), showing the positions of the heterozygous variants identified in the proband: c.1324C>T (p.Arg442Ter), classified as pathogenic in the original diagnostic report; c.1466A>C (p.Lys489Thr), classified as likely pathogenic; and c.82A>C (p.Ser28Arg), additionally detected. The DONSON gene is located at chromosome 21q22.11. (**B**) Comparison between the sequence configuration observed in the proband and the previously reported recurrent DONSON allele containing p.Ser28Arg, c.786-33A>G, and p.Lys489Thr. In the present case, p.Ser28Arg and p.Lys489Thr were detected, whereas c.786-33A>G was not detected; however, sequencing coverage of this intronic region was limited, precluding definitive exclusion. p.Arg442Ter was additionally present. The phase of the heterozygous variants was not established because parental segregation data were unavailable. (**C**) Transcript-specific NMDetective-B analysis of c.1324C>T (p.Arg442Ter) using the MANE Select transcript NM_017613.4 (ENST00000303071.10). The premature termination codon is not located in the last exon, is not within the terminal 50 nucleotides of the penultimate exon, is located more than 150 coding nucleotides from the translation-initiation codon and occurs in a coding exon of approximately 199 nucleotides, below the >407-nucleotide long-exon threshold. The resulting NMDetective-B score was 0.65, predicting susceptibility to nonsense-mediated mRNA decay. This represents an in silico prediction and was not experimentally confirmed in the proband.

**Figure 2:**
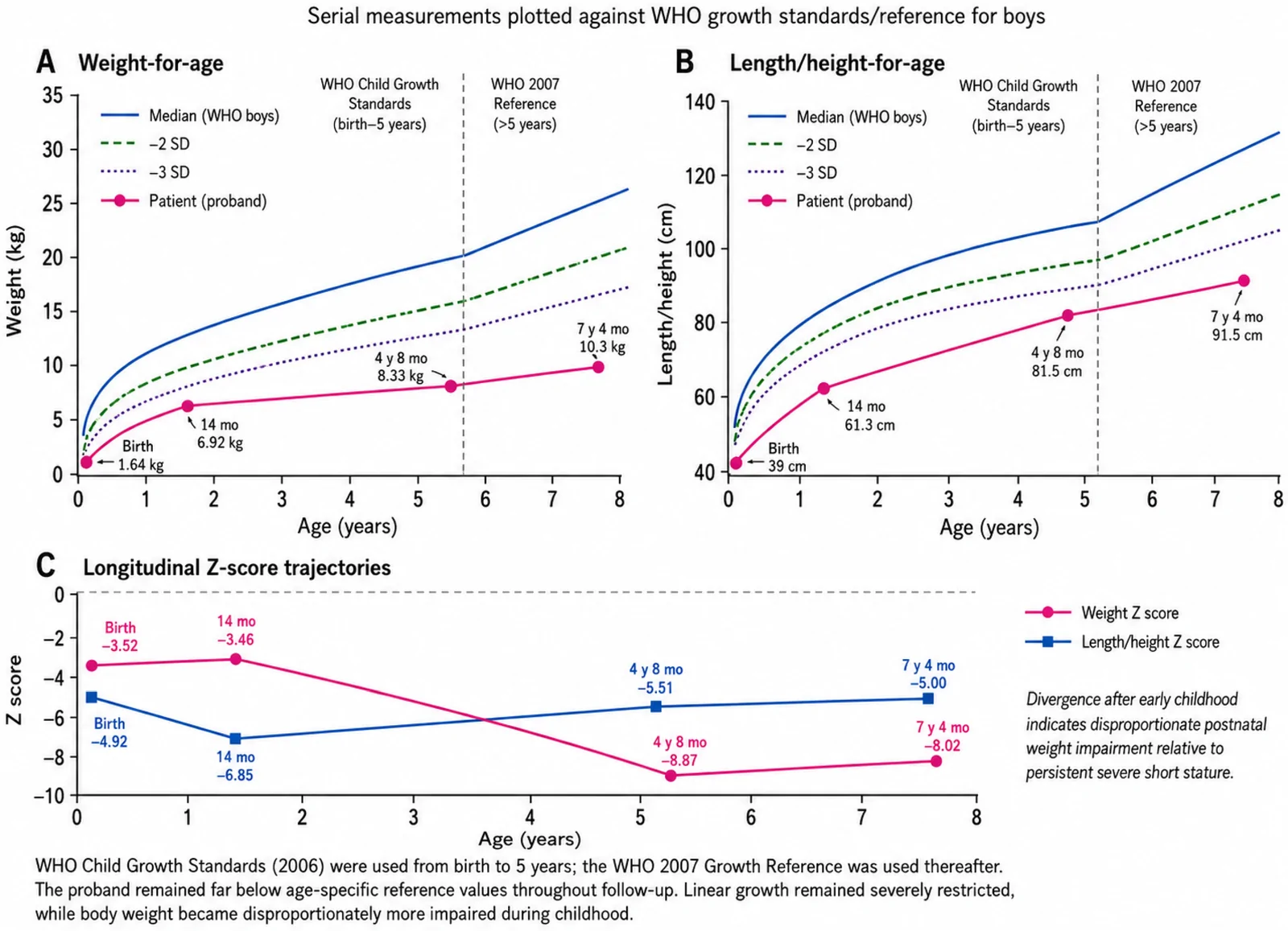
Longitudinal growth trajectories of the proband. (**A**) Weight-for-age measurements from birth through 7 years and 4 months plotted against World Health Organization reference values for boys. (**B**) Length/height-for-age measurements over the same follow-up period. WHO Child Growth Standards were used from birth to 5 years of age, followed by the WHO 2007 Growth Reference thereafter [7,8]. The proband remained markedly below age-specific reference values throughout follow-up. (**C**) Longitudinal comparison of weight and length/height Z-score trajectories. Linear growth was already severely impaired at birth and remained approximately 5 SD below the reference mean into school age, whereas weight became disproportionately more impaired during childhood, declining from approximately −3.52 SD at birth to −8.02 SD at 7 years and 4 months. This divergence supports persistent severe primordial short stature with a superimposed postnatal nutritional component. Minor short-interval fluctuations in recorded anthropometric values were retained as documented in the clinical record and may reflect routine measurement variability.

**Figure 3:**
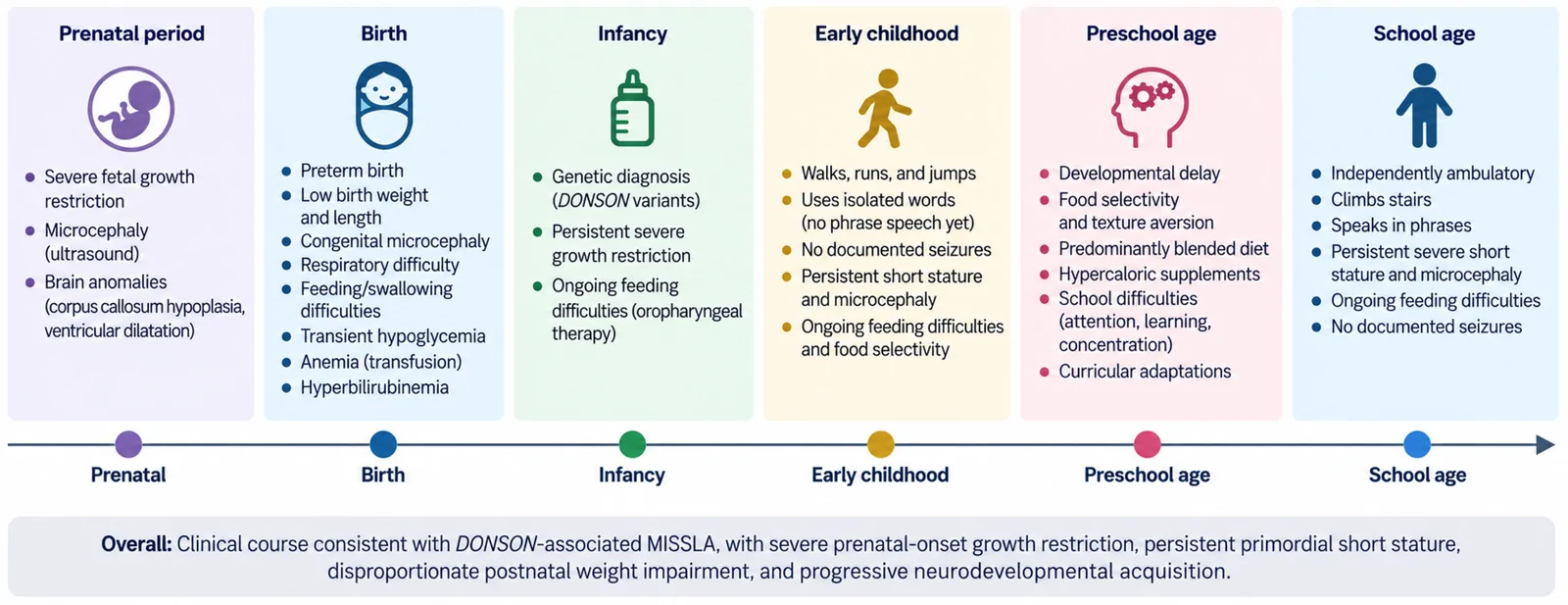
Longitudinal clinical timeline of the proband from the prenatal period to school age. The timeline summarizes the major clinical findings documented during longitudinal follow-up. Prenatal manifestations included severe fetal growth restriction, microcephaly, and structural brain abnormalities on prenatal imaging. At birth, the proband had low weight and length, congenital microcephaly, respiratory and feeding/swallowing difficulties, and transient neonatal complications including hypoglycemia, anemia, and hyperbilirubinemia. During infancy, DONSON variants were identified, with persistent severe growth restriction and feeding difficulties. Early childhood was characterized by delayed but progressive acquisition of motor and language skills, persistent short stature and microcephaly, and food selectivity. During the preschool period, developmental delay, texture aversion, nutritional support requirements, and learning difficulties requiring curricular adaptations were documented. By school age, the proband was independently ambulatory, could climb stairs and speak in phrases, while severe short stature, microcephaly, and feeding difficulties persisted. No seizures were documented during the reviewed follow-up. Overall, the longitudinal course is consistent with DONSON-associated MISSLA and demonstrates persistent prenatal-onset growth impairment with progressive neurodevelopmental acquisition.

The principal clinical, anthropometric, and genomic findings are summarized in Figure 1, Figure 2 and Figure 3 and Table 1 and Table 2. Longitudinal follow-up extended from the prenatal period through February 2026 and documented severe prenatal-onset growth restriction, congenital microcephaly, persistent profound short stature, progressive neurodevelopmental acquisition, prominent feeding difficulties, and comparatively mild skeletal involvement. Molecular evaluation identified co-occurring heterozygous DONSON variants, while transcript-specific computational analysis provided a testable prediction regarding the potential consequence of the truncating p.Arg442Ter variant.

### 3.1. Longitudinal Clinical Course

The proband was a male child born at approximately 37.3 weeks of gestation following a pregnancy complicated by severe fetal growth restriction. Prenatal ultrasonography demonstrated marked discordance between gestational age and fetal biometry, with an estimated fetal weight of approximately 1493 g shortly before delivery and severe reduction in head circumference. Fetal magnetic resonance imaging reported hypoplasia of the corpus callosum, bilateral atrial ventricular dilatation, and small arachnoid cysts, without major abnormalities of the brainstem or cerebellum. At birth, weight was 1.64 kg, length was 39 cm, and head circumference was 26.5 cm. The corresponding reported Z scores were approximately −3.52 for weight, −4.92 for length, and −5.58 for head circumference, documenting severe prenatal-onset growth restriction and congenital microcephaly. Neonatal complications included low birth weight for gestational age, transient hypoglycemia, respiratory difficulty, anemia requiring transfusion, hyperbilirubinemia, and feeding or swallowing difficulties.

Severe growth impairment persisted throughout childhood (Table 2; Figure 2). At approximately 14 months, weight was 6.92 kg and length was 61.3 cm, with a recorded height Z score of approximately −6.85. At 4 years and 8 months, weight was 8.33 kg (Z = −8.87), height was 81.5 cm (Z = −5.51), and body mass index Z score was −4.30. At 7 years and 4 months, weight was 10.3 kg (Z = −8.02), height was 91.5 cm (Z = −5.00), and body mass index Z score was −3.90. A subsequent measurement in February 2026 showed a weight of 10.2 kg and height of 92.0 cm. Linear growth therefore remained approximately 5 SD below the reference mean into school age, whereas body weight became disproportionately more impaired over time. This divergence occurred in parallel with persistent feeding difficulties, food selectivity, texture aversion, reduced oral intake, dependence on blended foods during early childhood, and use of nutritional supplementation.

Neurodevelopment was delayed but progressive. Early childhood assessments documented delayed acquisition of motor and language milestones, followed by independent walking and gradual development of expressive language. Later evaluations recorded the ability to walk, run, jump, perform basic fine-motor tasks, and progress from isolated words to phrase speech. A standardized developmental assessment yielded a developmental quotient of 39, recorded clinically as severe developmental delay. School-age assessments described difficulties with attention, learning, concentration, and autonomy that required curricular adaptations. Despite these impairments, continued acquisition of functional abilities was documented. By school age, the proband was independently ambulatory, could ascend and descend stairs, and communicated verbally using phrases. No seizures were documented during the reviewed follow-up.

Skeletal involvement was comparatively mild. Clinodactyly and bilateral flat feet were documented, whereas major limb-reduction abnormalities were not identified. Conventional cytogenetic analysis showed a 46,XY karyotype without a major numerical or structural chromosomal abnormality detectable by standard cytogenetics. Feeding and nutritional morbidity remained prominent throughout childhood, including early difficulties with sucking, swallowing, and mastication requiring oropharyngeal therapy, followed by marked food selectivity, texture aversion, occasional gagging or vomiting with certain foods, and the use of hypercaloric supplements and nutritional modules.

Longitudinal neuroimaging findings were not entirely concordant. Fetal and neonatal studies described hypoplasia of the corpus callosum, bilateral atrial ventricular dilatation interpreted as colpocephaly, and small arachnoid cysts. In contrast, a later cerebral MRI was interpreted as showing microcephaly and cortical thickening with reduced sulcation but described a morphologically normal corpus callosum and no colpocephaly. Because the original imaging studies were not centrally re-reviewed for the present case report, this discrepancy was retained as an unresolved longitudinal radiologic observation. The overall clinical course from fetal life through school age is summarized in Figure 3.

### 3.2. Molecular Findings

Clinical whole-exome sequencing identified two principal heterozygous DONSON variants: NM_017613.4:c.1324C>T (p.Arg442Ter) and NM_017613.4:c.1466A>C (p.Lys489Thr). In the original 2019 diagnostic report, p.Arg442Ter was classified as pathogenic and p.Lys489Thr as likely pathogenic. Heterozygous c.82A>C (p.Ser28Arg) was additionally detected. No clinically significant mitochondrial DNA variant associated with the phenotype was reported. The recurrent intronic c.786-33A>G variant, previously described as part of a recurrent p.Ser28Arg/c.786-33A>G/p.Lys489Thr allele, was reported as not detected. However, sequencing coverage of this intronic region was limited, and non-detection was therefore not considered equivalent to definitive molecular exclusion. The observed sequence configuration is summarized in Figure 1.

Parental testing had been recommended to establish segregation, but no formal molecular result demonstrating that p.Arg442Ter and p.Lys489Thr occur on opposite homologous chromosomes was identified in the available records. Compound heterozygosity was therefore not considered experimentally established. Accordingly, the molecular findings are reported as co-occurring heterozygous DONSON variants in a patient with a phenotype highly compatible with MISSLA, without assigning an unconfirmed trans configuration. The unresolved phase remains an important limitation of the molecular interpretation.

Contemporary database assessment provided additional context for p.Lys489Thr. In gnomAD v4.1, c.1466A>C (p.Lys489Thr) had an overall allele frequency of approximately 0.09%, indicating that population rarity alone was insufficient to support pathogenicity [11]. Contemporary ClinVar records included pathogenic or likely pathogenic interpretations for p.Lys489Thr in the context of MISSLA, whereas the original diagnostic laboratory classified the variant as likely pathogenic in 2019 [10]. The patient’s sequence configuration also differed from the previously reported recurrent DONSON allele containing p.Ser28Arg, c.786-33A>G, and p.Lys489Thr [2]. In the present proband, p.Ser28Arg and p.Lys489Thr were detected, p.Arg442Ter was additionally present, and c.786-33A>G was not detected in a region of limited sequencing coverage. Because phase was unresolved and the intronic variant could not be definitively excluded, these findings were not interpreted as establishing a novel haplotype. The comparison with previously reported p.Lys489Thr-positive cases is summarized in Table 1.

### 3.3. Transcript-Specific Prediction for p.Arg442Ter

The c.1324C>T substitution introduces a premature termination codon at amino acid position 442. Transcript-specific analysis using the MANE Select transcript NM_017613.4 (ENST00000303071.10) predicted that p.Arg442Ter is susceptible to nonsense-mediated mRNA decay. The premature termination codon occurs in coding exon 8 of 10, within an exon containing approximately 199 coding nucleotides, and is located more than 150 coding nucleotides downstream from the translation-initiation codon. It is not located in the last coding exon, does not fall within the terminal 50 nucleotides of the penultimate coding exon, is not start-proximal, and does not occur within a coding exon exceeding the 407-nucleotide long-exon threshold used by the NMDetective-B framework [12].

Accordingly, p.Arg442Ter followed the NMD-triggering terminal branch of the NMDetective-B decision model and yielded an NMD efficiency score of 0.65, corresponding to predicted susceptibility to nonsense-mediated mRNA decay [12]. Patient-derived RNA was not available, and transcript abundance, allelic expression, NMD activity, and DONSON protein abundance were not experimentally measured. The result therefore represents a transcript-specific computational prediction and should not be interpreted as experimental demonstration of NMD in the proband.

## 4. Discussion

This case provides a longitudinal clinical and genomic characterization of a child with a phenotype highly compatible with DONSON-associated microcephaly, short stature, and limb abnormalities syndrome (MISSLA). The principal contribution is the integration of clinical observations extending from fetal life through school age with variant-level genomic interpretation and a transcript-specific prediction for the truncating p.Arg442Ter variant. The phenotype was characterized by severe prenatal-onset growth restriction, congenital microcephaly, persistent profound short stature, disproportionate postnatal deterioration in body weight, developmental delay with continued acquisition of motor and language abilities, prominent feeding and nutritional difficulties, and comparatively mild skeletal involvement. At the molecular level, the patient carried co-occurring heterozygous p.Arg442Ter and p.Lys489Thr variants, with p.Ser28Arg additionally detected. These findings expand the longitudinal clinical documentation of surviving DONSON-associated disease but should not be interpreted as establishing a novel genotype–phenotype relationship or a patient-specific molecular mechanism.

The clinical course is consistent with the broad phenotypic spectrum previously associated with biallelic DONSON variation. Severe prenatal and postnatal growth restriction, microcephaly, neurodevelopmental impairment, and variable skeletal abnormalities have been repeatedly described in affected individuals [2,3]. The present case is notable for the persistence of severe linear-growth restriction from birth into school age, while body weight became disproportionately more impaired during childhood. Length was already approximately 4.92 SD below the reference mean at birth and height remained approximately 5 SD below the mean at 7 years and 4 months, whereas weight declined from approximately −3.52 SD at birth to −8.02 SD at the same school-age assessment. This divergence suggests that the genetically determined primordial growth disorder was accompanied by an additional postnatal nutritional burden. The documented feeding difficulties, texture aversion, restricted oral intake, dependence on blended foods, and need for nutritional supplementation provide clinical support for this interpretation, although the relative contributions of genetic and nutritional factors cannot be quantified from a single retrospective case.

Neurodevelopmental impairment was substantial but not static. The child showed delayed acquisition of motor and language milestones but continued to gain functional abilities throughout follow-up, ultimately achieving independent ambulation, stair climbing, and phrase speech. This longitudinal observation is clinically relevant because cross-sectional descriptions may emphasize developmental impairment without capturing continued acquisition of abilities over time. Skeletal involvement was comparatively mild, consisting primarily of clinodactyly and bilateral flat feet without major limb-reduction defects. Such findings remain compatible with DONSON-associated disease because the skeletal phenotype is heterogeneous and can range from relatively mild abnormalities to severe micromelia and Meier–Gorlin-like presentations [5]. The present case therefore reinforces the importance of interpreting the phenotype as an integrated clinical spectrum rather than requiring the complete expression of the most severe manifestations reported in the literature.

The neuroimaging findings also illustrate the limitations of retrospective longitudinal assessment. Fetal and neonatal studies described corpus-callosum hypoplasia and ventricular atrial dilatation interpreted as colpocephaly, whereas a later MRI described a morphologically normal corpus callosum and no colpocephaly, together with microcephaly and reduced sulcation. Because the original imaging examinations were not centrally re-reviewed, these differences cannot be resolved retrospectively. Reporting the discordant interpretations is preferable to selecting one historical report as definitive, particularly in an ultra-rare disorder in which neuroimaging findings may influence phenotypic characterization.

The molecular configuration requires similarly cautious interpretation. p.Lys489Thr has previously been reported as part of a recurrent DONSON allele containing p.Ser28Arg, c.786-33A>G, and p.Lys489Thr [2]. In the present patient, p.Ser28Arg and p.Lys489Thr were detected, whereas c.786-33A>G was reported as not detected and p.Arg442Ter was additionally present. However, sequencing coverage of the intronic region containing c.786-33A>G was limited, preventing definitive exclusion of that variant. More importantly, no parental segregation result demonstrating that p.Arg442Ter and p.Lys489Thr occur on opposite homologous chromosomes was available. The present findings therefore establish co-occurrence of heterozygous DONSON variants but do not establish confirmed compound heterozygosity. This distinction is particularly important because MISSLA is predominantly associated with biallelic DONSON variation [3]. The strong phenotypic concordance supports a DONSON-related diagnosis, but phenotype alone cannot determine chromosomal phase.

Interpretation of p.Lys489Thr also requires integration of several forms of evidence rather than reliance on population rarity. The variant has an overall allele frequency of approximately 0.09% in gnomAD v4.1 [11], indicating that it should not be described as a private or exceptionally rare substitution. Contemporary ClinVar records include pathogenic or likely pathogenic interpretations in the context of MISSLA [10], while the original 2019 diagnostic report classified the variant as likely pathogenic. Importantly, Reynolds et al. experimentally evaluated p.Lys489Thr and reported reduced DONSON protein abundance and incomplete correction of spontaneous replication-fork stalling [2]. These findings provide variant-specific functional support, but they were obtained in previously studied experimental systems and should not be presented as patient-specific functional evidence in the present case. Similarly, the co-occurrence of p.Ser28Arg does not imply that both substitutions contribute equally to dysfunction; previous experiments showed a more evident effect for p.Lys489Thr than for p.Ser28Arg in the evaluated setting [2].

The p.Arg442Ter variant provides a distinct interpretive challenge because it introduces a premature termination codon. Other truncating DONSON variants in the same general C-terminal region, including p.Lys419Ter and p.Gln428Ter, have been reported in individuals with microcephalic primordial dwarfism [2], providing precedent for disease-associated truncating alleles in this portion of the gene. In the present analysis, NMDetective-B predicted that p.Arg442Ter is susceptible to nonsense-mediated mRNA decay, with a score of 0.65 [12]. The prediction is biologically plausible because the premature termination codon does not meet the transcript-position criteria associated with NMD escape in the MANE Select transcript NM_017613.4. Nevertheless, this finding remains computational. Patient-derived RNA was unavailable, and transcript abundance, allelic expression, NMD activity, and DONSON protein abundance were not measured. It would therefore be inappropriate to state that the mutant transcript undergoes NMD in this patient. The appropriate interpretation is that p.Arg442Ter is predicted to be NMD-susceptible and that this prediction provides a testable hypothesis for future functional studies.

This distinction between published biological mechanisms and patient-specific evidence is essential. DONSON has established roles in DNA-replication initiation and assembly of the CDC45–MCM2-7–GINS replicative helicase [6], and previous experimental studies have demonstrated replication-fork instability and impaired genome maintenance in disease-associated DONSON deficiency [2]. These mechanisms provide a coherent biological framework for severe prenatal growth restriction and microcephaly. However, replication-fork dynamics, DNA damage, CMG assembly, cell proliferation, RNA abundance, and DONSON protein expression were not evaluated in the present patient. The current case therefore presents longitudinal clinical characterization and variant-level computational interpretation rather than direct demonstration of a new molecular mechanism.

Several limitations should be considered. First, this is a single-patient case report, and the observed natural history cannot be generalized to all individuals with DONSON-associated disease or used to establish variant-specific genotype–phenotype correlations. Second, parental segregation and experimental phasing were unavailable, preventing confirmation that the principal variants occur in trans. Third, the NMD prediction was not experimentally validated because patient-derived RNA and protein were unavailable. Fourth, limited coverage prevented definitive exclusion of c.786-33A>G. Fifth, some longitudinal information was derived from routine clinical records rather than prospectively standardized research assessments, and inconsistencies were present among historical anthropometric and neuroimaging reports. Finally, available protein annotation does not define a well-established discrete functional subdomain specifically encompassing residues 442 and 489; mechanistic interpretation based solely on positional protein-domain assignment would therefore be speculative.

Parental molecular testing is the most immediate step needed to strengthen the genomic interpretation. Demonstrating that the principal variants were inherited from different parents would establish a trans configuration, whereas a cis configuration would require substantial reassessment of the proposed molecular diagnosis. If biological material becomes available, allele-specific RNA studies could directly test whether the p.Arg442Ter-containing transcript undergoes NMD, and quantitative protein studies could determine whether altered transcript abundance is accompanied by reduced DONSON expression. Additional functional experiments could evaluate replication-related cellular phenotypes, but such effects should remain hypotheses until directly demonstrated in patient-derived material. Continued longitudinal follow-up of growth, nutrition, pubertal development, neurodevelopment, language, learning, and functional independence may also help define the natural history of surviving DONSON-associated MISSLA and clarify the contribution of potentially modifiable nutritional morbidity to the overall phenotype.

## 5. Conclusions

This case extends the longitudinal clinical and genomic characterization of DONSON-associated MISSLA from the prenatal period through school age. The proband showed persistent severe prenatal-onset growth restriction and congenital microcephaly, progressive neurodevelopmental acquisition despite substantial developmental impairment, prominent feeding and nutritional difficulties, and comparatively mild skeletal involvement. Molecular analysis identified co-occurring heterozygous DONSON p.Arg442Ter and p.Lys489Thr variants, with p.Ser28Arg additionally detected, but the absence of parental segregation data prevents confirmation that the principal variants occur in trans. Transcript-specific NMDetective-B analysis predicted that p.Arg442Ter is susceptible to nonsense-mediated mRNA decay, providing a testable molecular hypothesis rather than direct evidence of transcript degradation in the patient. The lack of patient-derived RNA, protein studies, and functional replication assays precludes definitive attribution of the phenotype to a specific molecular mechanism. The principal value of this case lies in the integration of long-term phenotypic follow-up with careful variant-level interpretation while explicitly distinguishing documented patient findings from computational predictions and previously published functional evidence. Future parental segregation and patient-derived functional studies will be important to refine the molecular interpretation.

## Figures and Tables

**Table 1:** Published DONSON p.Lys489Thr-positive cases and comparison with the present proband.

Source/Grouped Report	p.Lys489Thr Context	Additional Reported DONSON Variant(s)	Broad Reported Phenotype
Reynolds et al. [2], two affected individuals	Present within recurrent p.Ser28Arg/c.786-33A>G/p.Lys489Thr allele	c.1251_1256delCTCTAA, p.Asn417_Ser418del	Microcephalic primordial dwarfism/MISSLA spectrum
Reynolds et al. [2]	Present within recurrent p.Ser28Arg/c.786-33A>G/p.Lys489Thr allele	c.877C>T, p.Arg293Ter	Microcephalic primordial dwarfism/MISSLA spectrum
Reynolds et al. [2]	Present within recurrent p.Ser28Arg/c.786-33A>G/p.Lys489Thr allele	c.1254dupT, p.Lys419Ter	Microcephalic primordial dwarfism/MISSLA spectrum
Reynolds et al. [2]	Present within recurrent p.Ser28Arg/c.786-33A>G/p.Lys489Thr allele	c.1686dupT, p.Asn563Ter	Microcephalic primordial dwarfism/MISSLA spectrum
Reynolds et al. [2]	Present within recurrent p.Ser28Arg/c.786-33A>G/p.Lys489Thr allele	Additional missense DONSON variants reported	Microcephalic primordial dwarfism/MISSLA spectrum
Reynolds et al. [2], two affected individuals	Present within recurrent p.Ser28Arg/c.786-33A>G/p.Lys489Thr allele	c.1282C>T, p.Gln428Ter	Microcephalic primordial dwarfism/MISSLA spectrum
Vaseghi et al. [14]	p.Lys489Thr detected	p.Ser28Arg additionally detected	Fetal multiple-congenital-anomaly phenotype
Present proband	p.Lys489Thr detected	c.1324C>T (p.Arg442Ter); p.Ser28Arg additionally detected; c.786-33A>G not detected in a region of limited coverage	Severe prenatal-onset growth restriction, congenital microcephaly, developmental impairment, feeding difficulties, mild limb involvement, and survival into school age

Published individuals were grouped when they shared the same reported molecular configuration to reduce unnecessary individual-level detail and emphasize variant-level comparison. In the Reynolds cohort, p.Lys489Thr was generally reported within a recurrent allele containing p.Ser28Arg, c.786-33A>G, and p.Lys489Thr [2]. In the present proband, p.Ser28Arg and p.Lys489Thr were detected, whereas c.786-33A>G was not detected; however, coverage of this intronic region was limited, preventing definitive exclusion. Parental segregation data were unavailable, and the phase of the heterozygous variants was therefore not established. The present case is consequently described as carrying co-occurring heterozygous DONSON variants rather than confirmed compound heterozygosity.

**Table 2:** Longitudinal anthropometric trajectory of the proband.

Clinical Stage	Weight	Length/Height	Available Z Scores	Main Clinical Interpretation
Birth	1.64 kg	39 cm	W −3.52; L −4.92	Severe prenatal-onset growth restriction; HC 26.5 cm
Early infancy (~3 months)	2.64 kg	46.5 cm	—	Persistent marked postnatal growth deficit
Late infancy (~14 months)	6.92 kg	61.3 cm	W −3.46; H −6.85	Severe persistent short stature
Preschool follow-up 1	8.33 kg	81.5 cm	W −8.87; H −5.51; BMI −4.30	Profound growth restriction with marked weight impairment
Preschool follow-up 2	8.60 kg	82.4 cm	W −8.70; H −5.53; BMI −3.33	Persistent severe short stature and low body weight
Preschool follow-up 3	8.9 kg	84.0 cm	W −8.44; H −5.31; BMI −3.49	Persistent short stature with nutritional compromise
Preschool follow-up 4	8.8 kg	83.5 cm	—	Persistently low body mass and poor weight gain
School-age follow-up 1	10.3 kg	91.5 cm	W −8.02; H −5.00; BMI −3.90	Persistent severe short stature with disproportionate weight impairment
School-age follow-up 2	10.2 kg	92.0 cm	—	Continued profound growth impairment

Calendar dates were removed to protect patient confidentiality; measurements are presented in chronological clinical order using broad developmental stages. W, weight-for-age Z score; L, length-for-age Z score; H, height-for-age Z score; BMI, body mass index Z score; HC, head circumference. Reported Z scores were retained from the clinical record when available. Minor short-interval fluctuations in recorded weight or height were preserved as documented and may reflect routine clinical measurement variability. WHO Child Growth Standards were used for early childhood and the WHO 2007 Growth Reference thereafter [7,8]. The longitudinal pattern demonstrates persistent severe linear-growth restriction together with disproportionately greater postnatal impairment of body weight.

## Data Availability

The clinical data supporting this report are contained within the patient’s confidential medical record and are not publicly available. De-identified data relevant to the conclusions of this report may be provided where permitted by institutional and ethical requirements.

## Abbreviations

The following abbreviations are used in this manuscript:

BMI	body mass index
CIRA	Institutional Committee for Academic Review
CMG	CDC45–MCM2-7–GINS replicative helicase complex
DNA	deoxyribonucleic acid
gnomAD	Genome Aggregation Database
GRCh37	Genome Reference Consortium Human Build 37
HGVS	Human Genome Variation Society
MANE	Matched Annotation from NCBI and EMBL-EBI
MISSLA	microcephaly, short stature, and limb abnormalities syndrome
NCBI	National Center for Biotechnology Information
NMD	nonsense-mediated mRNA decay
OMIM	Online Mendelian Inheritance in Man
PTC	premature termination codon
RNA	ribonucleic acid
SD	standard deviation
WES	whole-exome sequencing
WHO	World Health Organization
